# Investigating the pavement performance and aging resistance of modified bio-asphalt with nano-particles

**DOI:** 10.1371/journal.pone.0238817

**Published:** 2020-09-04

**Authors:** Jiaolong Ren, Guangyuan Zang, Siyuan Wang, Jun Shi, Yuanyuan Wang

**Affiliations:** 1 School of Civil and Architectural Engineering, Shandong University of Technology, Zibo, Shandong, China; 2 School of Civil Engineering and Architecture, Hubei University of Arts and Science, Xiangyang, Hubei, China; Mirpur University of Science and Technology, PAKISTAN

## Abstract

Bio-asphalt binders have been proposed as replacements for traditional asphalt binders, owing to advantages such as environmental protection, low costs, and abundant resources. However, a limitation of bio-asphalt binders is that their high-temperature performance is not suitable for pavement construction. In recent years, nano-particles have been widely used to improve the pavement performance of asphalt binders, particularly the high-temperature performance. Thus, the nano-particles might also provide a positive modified effect on the high-temperature performance of bio-asphalt binders. Based on this, five types of nano-particles including SiO_2_, CaCO_3_, TiO_2_, Fe_2_O_3_, and ZnO are selected for the preparation of modified bio-asphalt binders, using different dosages of nano-particles and bio-oil. The high- and low-temperature performances, aging resistance, workable performance, and water stability of the nano-modified bio-asphalt binders and mixtures are investigated. The results reveal that, the high-temperature performance and aging resistance of the nano-modified bio-asphalt binders and mixtures are improved at increased nano-particle dosages, whereas their low-temperature performance is slightly weakened. The effects of the nano-particles on the workable performance and water stability are insignificant.

## Introduction

Asphalt mixture is one of the most important materials in pavement engineering. The demand for asphalt binder continues to grow with large-scale pavement construction and maintenance; however, the reserves of asphalt binder are decreasing worldwide, owing to the exhaustion of petroleum resources. Hence, there is a need to study alternative materials to reduce the consumption of asphalt binder, and to meet the technical requirements for pavement construction.

Bio-oil has been introduced as a replacement for traditional asphalt materials, owing to its advantages in environmental protection, low costs, and abundant resources. The physicochemical characteristics and pavement properties of different types of bio-oil modified asphalt binders have been extensively analyzed in recent years [1–12]. The results have revealed that bio-oil modified asphalt binders can provide better low-temperature performance than traditional asphalt binders, as the bio-oil alleviates the thermal stress more easily [13]. However, the high-temperature stabilities of bio-oil modified asphalt binders are unsatisfactory. Fini et al. [1] discovered that a bio-asphalt binder might decrease the high-temperature grade of asphalt binders. Gao et al. [2], Sun et al. [3,4], Yong et al. [5], Wang et al. [6], and Dhasmana et al. [7] found that the bio-oils weakened the stiffness and temperature stability of asphalt binders at high temperatures. Mirhosseini et al. [8] indicated that the high-temperature performance of a bio-asphalt binder could be adversely affected owing to the softening effect of bio-oil. Lei et al. [9,10] demonstrated that at high temperatures, asphalt mastics modified with bio-oils exhibited relatively low rheological performance. Yang et al. [11] and Mohammad et al. [12] discovered that the rutting resistance of a bio-asphalt mixture was not better than that of a base asphalt mixture. Hence, it is important to develop a technology for enhancing the high-temperature performance of bio-oil modified asphalt binders.

In recent years, nanomaterials (i.e., materials with at least one dimension within 1–100 nm) have exhibited novel properties owing to the nature of their large specific surface areas, high surface free energies, and satisfactory dispersion abilities, attracting attention from engineers. These unique properties of nano-materials can provide a bridge between macroscopic materials and molecular structures to improve the mechanical, thermodynamic, and chemical properties of a material. Existing studies show that nano-materials can successfully modify asphalt binders to improve the pavement performance, especially the high-temperature performance [14–25]. Particularly, the effects from the nano-particles are more significant, owing to the much larger surface area per unit mass as a nanomaterial decreases in size. For instance, nano-CaCO_3_ particles [14,15], nano-ZnO particles [16,17], nano-TiO_2_ particles [18,19]_,_ nano-SiO_2_ particles [20,21], and nano-Fe_2_O_3_ particles [22,23] have been widely investigated for the preparation of nano-modified asphalt binders and have exhibited satisfactory results. It is reasonable to speculate that nano-particles could also be used to improve the high-temperature performance of bio-asphalt binders.

However, there is relatively little literature addressing the relationship between nano-particles and bio-asphalt binders. Thus, this study conducts a series of traditional tests and rolling thin film oven tests (RTFOTs) for different modified asphalt binders with nano-particles to examine their high-temperature performance, low-temperature performance, and aging resistance, and to evaluate the most effective type of nano-particles.

## Materials and methods

### Materials

#### (1) Base asphalt binder

The 70# base asphalt binder (AH-70) manufactured in Shandong Province of China is used in laboratory tests. The technical properties are shown in Table 1.

**Table 1:** Technical properties of the base asphalt binder.

25°C penetration (0.1mm)	Softening point (°C)	10°C ductility (cm)	15°C ductility (cm)	60°C viscosity (Pa.s)
67.4	47.4	17.2	>100	316

#### (2) Nano-particles

Five types of nano-particles including SiO_2_, CaCO_3_, TiO_2_, Fe_2_O_3_, and ZnO are selected to prepare the modified asphalt binders. The characteristics of the selected nano-particles are listed in Table 2. These nano-particles are provided by Hangzhou Jialu Transportation Technology Co. LTD. The technical properties of modified asphalt binders with different nano-particles are shown in Table 3.

**Table 2:** Particle size and specific surface area of the selected nano-particles.

Type	Particle size (nm)	Specific surface area (m^2^/g)	Volume density (g/cm^3^)	Purity (%)	Appearance
SiO_2_	15	600	0.21	99.8	White powder
CaCO_3_	30	60	0.68	99.0	White powder
TiO_2_	35	110	0.35	99.9	White powder
Fe_2_O_3_	20	210	0.51	99.9	Red powder
ZnO	30	24	1.30	99.9	White powder

**Table 3:** Technical properties of modified asphalt binders with different nano-particles.

Type		25°C penetration (0.1mm)	Softening point (°C)	10°C Ductility (cm)	15°C ductility (cm)	135°C viscosity (Pa·s)
AH-70+	1% SiO_2_	61.1	55.5	10.6	>100	1.176
	1% CaCO_3_	66.2	51.8	12.6	>100	1.064
	1% TiO_2_	64.6	52.8	11.9	>100	1.085
	1% Fe_2_O_3_	62.6	51.9	11.3	>100	1.116
	1% ZnO	64.9	51.5	11.5	>100	1.061

#### (3) Bio-oil

The bio-oil is obtained through the decomposition of saw dust using pyrolysis. The bio-oil is provided by Hangzhou Jialu Transportation Technology Co. LTD. The basic technical parameters are listed in Table 4. The technical properties of bio-asphalt binders are shown in Table 5.

**Table 4:** Technical parameters of the bio-oil.

Mass fraction of compositions (%)					Density (g/cm^3^)	PH	135°C viscosity (Pa·s)
C	H	O	N	S			
48.16	6.62	39.39	0.11	0.97	1.06	2.9	0.826

**Table 5:** Technical properties of bio-asphalt binders.

Type		25°C penetration (0.1mm)	Softening point (°C)	5°C ductility (cm)	135°C viscosity (Pa.s)
AH-70 +	3% bio-oil	80.7	40.6	21.1	0.531
	5% bio-oil	103.5	37.9	40.6	0.344
	7% bio-oil	--	35.8	55.9	--

### Experimental methods

According to the Chinese technical specification “*Technical specification for construction of highway asphalt pavements (JTG F40-2004)*” [26], the 25°C penetration is used to confirm the grading level of the asphalt binder. The softening point is used to evaluate the high-temperature performance of the asphalt binder. The 5°C ductility is used to evaluate the low-temperature performance of the asphalt binder. The 135°C dynamic viscosity is used to evaluate the workable performance of the asphalt binder, and it must be lower than 3.0 Pa·s. The workable performance refers to the ability of the asphalt binder to mix with aggregates conveniently during the preparation process of asphalt mixture to ensure the uniformity of the asphalt mixture. The mass loss, 25°C residual penetration ratio and 5°C ductility after aging, obtained via RTFOTs, are used to evaluate the aging resistance of the asphalt binder.

The dynamic stability, obtained via rutting test, is used to verify the high-temperature stability of the asphalt mixture. The fracture strain, obtained via bending test at -10°C, is used to verify the low-temperature crack resistance of the asphalt mixture. The residual stability, obtained via immersing Marshall test, is used to verify the water stability of the asphalt mixture. The water stability refers to the ability of the asphalt mixture to resist damages (e.g., loosening and pit slot) from water erosion.

All the tests are conducted in accordance with the Chinese test standard “*Standard test methods of bitumen and bituminous mixtures for highway engineering* (*JTG E20-2011*)” [27].

### Preparation of the modified bio-asphalt binders

Based on the principle of dissolution in a similar material structure, it is easy to mix the bio-oil with the base asphalt binder completely. However, owing to the large specific surface area and high surface energy, nano-materials have a great inclination to agglomerate and form secondary particles. This agglomeration behavior causes negative modification effects. Hence, it is necessary to disperse the nanomaterials uniformly to overcome the agglomeration problem. In this study, in accordance with our previous study [28,29], a high-temperature, high-speed shearing method is adopted to address the agglomeration issue.

The modified asphalt binders that use nano-particles and bio-oil are manufactured as follows. The bio-oil and base asphalt binder are mixed, thereby forming the bio-asphalt binder. The total mass of the bio-oil and base asphalt binder is 500 g and is kept constant. Then, the nano-modified bio-asphalt binders are prepared after adding and mixing nano-particles via 10 min of high-speed shearing (5000 r/min) at 120°C.

### Pavement properties of modified bio-asphalt binders

The test results of 25°C penetration, softening point, 5°C ductility, and 135°C dynamic viscosity of the different asphalt binders are shown in Table 6.

**Table 6:** Technical properties of different asphalt binders.

Type			25°C penetration (0.1mm)	Softening point (°C)	5°C ductility (cm)	135°C viscosity (Pa.s)
AH-70			67.4	47.4	0	0.680
AH-70 +	3% bio-oil		80.7	40.6	21.1	0.531
	5% bio-oil		103.5	37.9	40.6	0.344
	7% bio-oil		--	35.8	55.9	--
AH-70 +	3%bio-oil +	0.2%SiO_2_	70.1	45.3	16.4	0.579
		0.5%SiO_2_	67.1	47.7	15.5	0.638
		0.8%SiO_2_	64.6	48.9	14.6	0.671
	5%bio-oil +	0.2%SiO_2_	82.8	43.7	30.1	0.568
		0.5%SiO_2_	76.7	45.3	24.2	0.630
		0.8%SiO_2_	70.1	46.1	19.9	0.645
	7%bio-oil +	0.2%SiO_2_	92.1	41.9	43.7	0.398
		0.5%SiO_2_	87.6	44.1	38.9	0.462
		0.8%SiO_2_	83.8	44.6	35.8	0.484
AH-70 +	3%bio-oil +	0.2%CaCO_3_	78.2	42.1	17.3	0.546
		0.5%CaCO_3_	73.1	44.4	16.3	0.602
		0.8%CaCO_3_	68.7	45.5	15.3	0.633
	5%bio-oil +	0.2%CaCO_3_	92.4	40.6	32.4	0.536
		0.5%CaCO_3_	85.6	42.2	26.0	0.594
		0.8%CaCO_3_	78.2	42.9	21.4	0.608
	7%bio-oil +	0.2%CaCO_3_	102.8	39.3	46.3	0.375
		0.5%CaCO_3_	97.8	41.0	41.1	0.436
		0.8%CaCO_3_	93.5	41.5	37.8	0.456
AH-70 +	3%bio-oil +	0.2%TiO_2_	75.7	42.6	15.8	0.565
		0.5%TiO_2_	70.6	44.8	14.9	0.623
		0.8%TiO_2_	66.5	46.0	14.1	0.655
	5%bio-oil +	0.2%TiO_2_	89.4	41.1	28.4	0.554
		0.5%TiO_2_	82.8	42.6	22.8	0.615
		0.8%TiO_2_	75.7	43.4	18.8	0.630
	7%bio-oil +	0.2%TiO_2_	99.5	39.6	41.8	0.388
		0.5%TiO_2_	94.6	41.5	37.3	0.451
		0.8%TiO_2_	90.5	41.9	34.3	0.472
AH-70 +	3%bio-oil +	0.2%Fe_2_O_3_	71.2	41.9	16.1	0.570
		0.5%Fe_2_O_3_	66.1	44.1	15.2	0.628
		0.8%Fe_2_O_3_	62.5	45.2	14.3	0.660
	5%bio-oil +	0.2%Fe_2_O_3_	84.0	40.4	29.2	0.559
		0.5%Fe_2_O_3_	77.9	42.0	23.5	0.620
		0.8%Fe_2_O_3_	71.2	42.6	19.3	0.635
	7%bio-oil +	0.2%Fe_2_O_3_	93.5	38.9	42.7	0.392
		0.5%Fe_2_O_3_	88.9	40.8	38.0	0.455
		0.8%Fe_2_O_3_	85.1	41.3	35.0	0.476
AH-70 +	3%bio-oil +	0.2%ZnO	72.6	41.4	16.3	0.544
		0.5%ZnO	67.6	43.6	15.4	0.600
		0.8%ZnO	63.8	44.7	14.5	0.631
	5%bio-oil +	0.2%ZnO	85.8	39.9	29.8	0.534
		0.5%ZnO	79.5	41.7	24.0	0.592
		0.8%ZnO	72.6	42.2	19.7	0.606
	7%bio-oil +	0.2%ZnO	95.4	38.1	43.4	0.374
		0.5%ZnO	90.8	40.3	38.6	0.434
		0.8%ZnO	86.8	41.0	35.6	0.455

**Fig 1:**
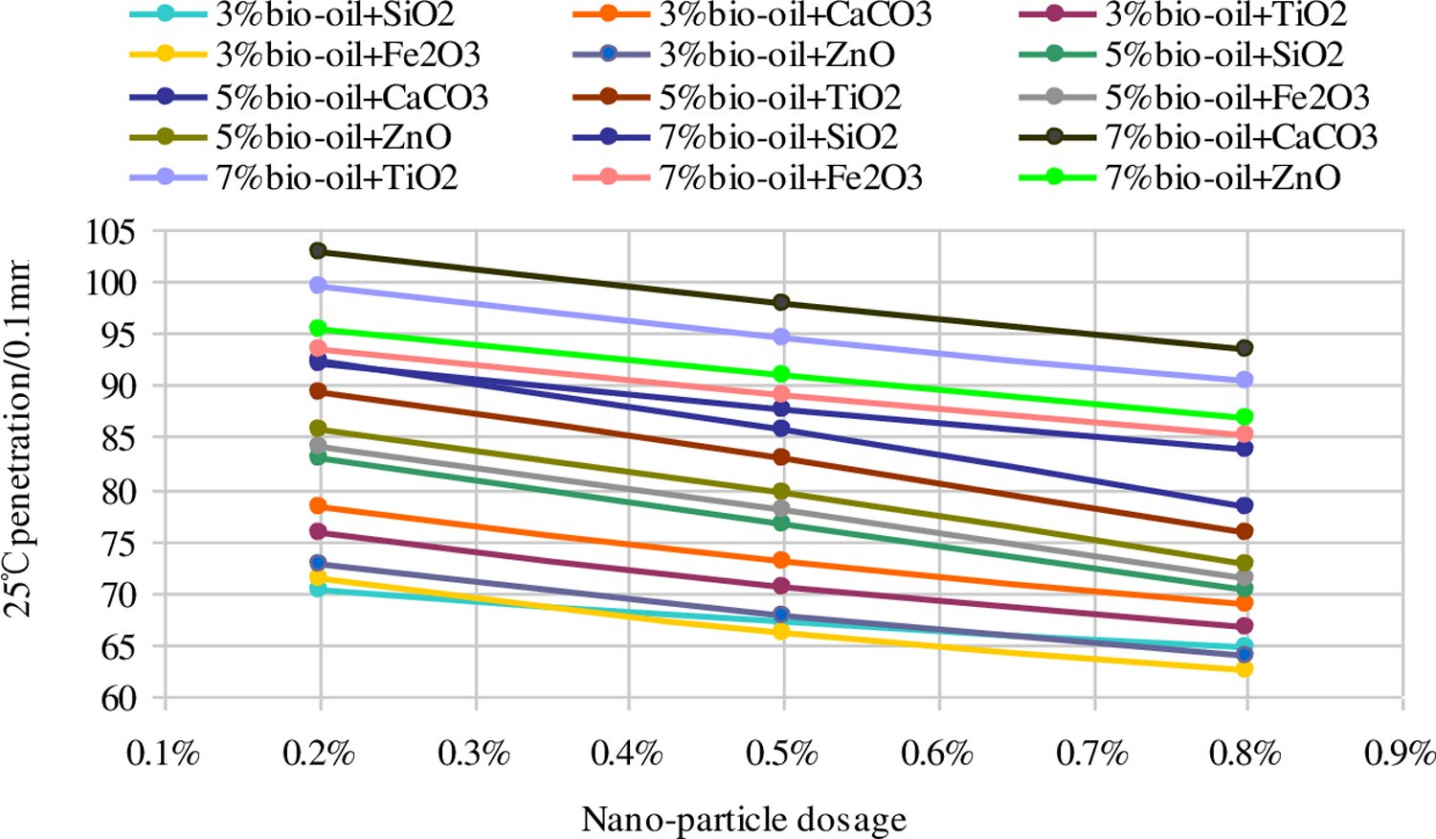
Trend of 25°C penetration with different nano-particle dosages.

As shown in Table 6, it can be found that:

Fig 1 displays the trend of 25°C penetration with different nano-particle dosages. The 25°C penetrations of the nano-modified bio-asphalt binders decrease linearly as the nano-particle dosages increase. Moreover, compared to the bio-asphalt binder, the decrease in the 25°C penetration is the most when nano-SiO_2_ is used, and is the least when nano-CaCO_3_ is used.

The softening points of the nano-modified bio-asphalt binders increase as the nano-particle dosages increase, showing the positive effect of the nano-particles on the high-temperature performance of a bio-asphalt binder. However, the modified effects of different nano-particles become insignificant with the increased nano-particle dosages, particularly when the dosage is larger than 0.5%. The trends tend to flatten with increased nano-particle dosages, and there is less of a difference with a change of the nano-particles. Among the tested five types of nano-particles, nano-SiO_2_ has the best modified effect on the softening point and nano-ZnO is the worst. The difference is owing to the difference in the specific surface areas, implying that nano-particles with a higher specific surface area can provide a better modifying effect on the high-temperature performance of nano-modified bio-asphalt binders.

An asphalt binder can be treated as a mix of three types of distributed systems: a matrix phase, dispersed phase, and bee-like structure. The softening point of the asphalt binder increases as the proportion of the matrix phase increases, and the 5°C ductility and 25°C penetration increase as the proportion of the dispersed phase increase [30–35]. Evidently, the proportion of the dispersed phase in the asphalt binder increases with the addition of bio-oil [30]. Hence, the 5°C ductility and 25°C penetration of the bio-asphalt binder increase with an increased proportion of bio-oil, and the softening point decreases accordingly. In addition, owing to the large specific surface area, the nano-particles fuse with the dispersed phase and bee-like structure well [31,32] and accelerate the transformation from the dispersed phase and bee-like structure to the matrix phase [31,33] (particularly for the bee-like structure). This makes the asphalt binder less prone to deformation, softening, and flowing, so that the nano-particles can improve the softening point of the bio-asphalt binder and reduce the 25°C penetration. Moreover, the aforementioned action of the nano-particles is more significant when the specific surface area of nano-particles is increased. Hence, nano-SiO_2_ displays the best modification effect for the bio-asphalt binder.

**Fig 2:**
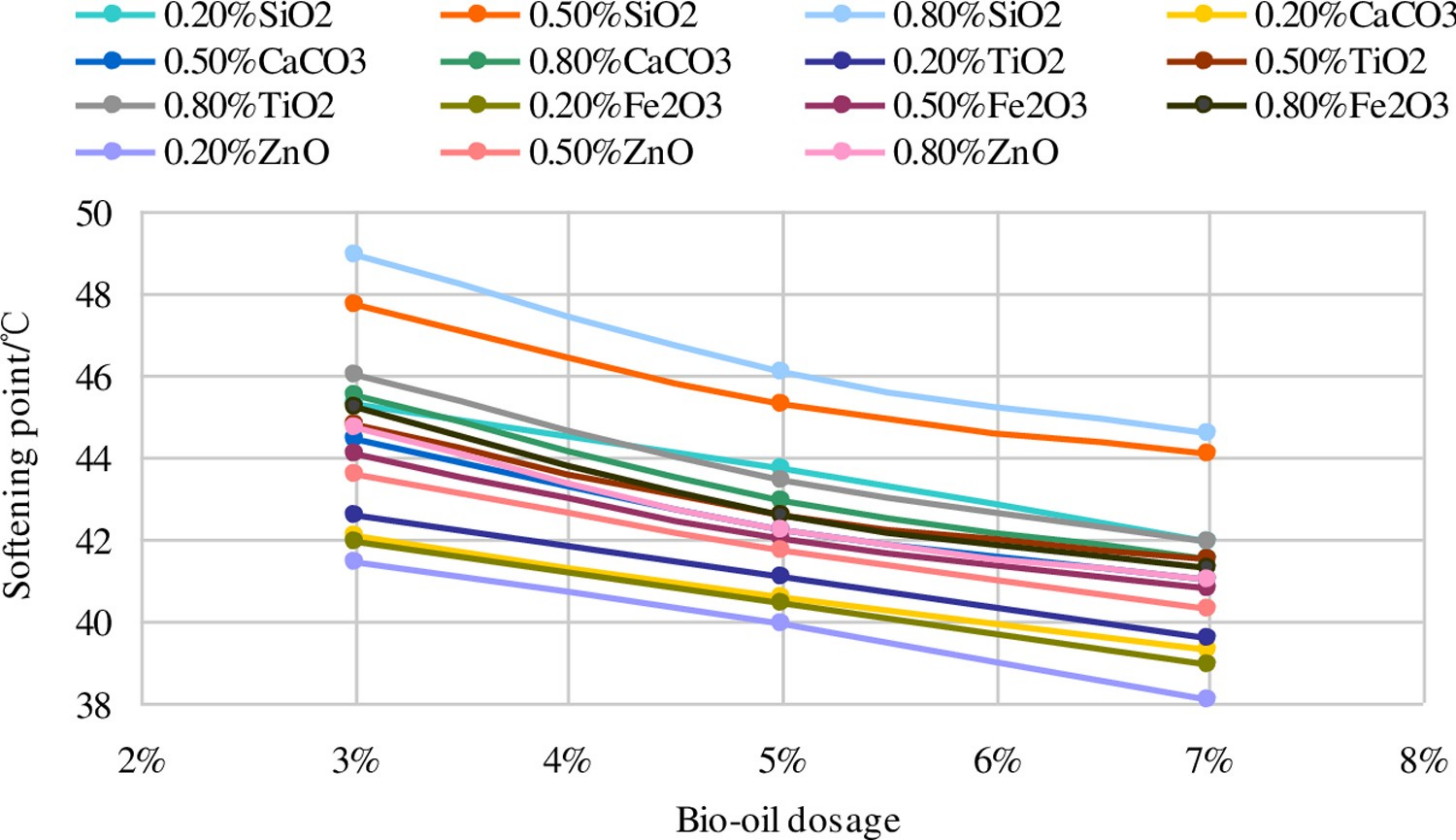
Trend of softening point with different bio-oil dosages.

Fig 2 displays the trend of softening point with different bio-oil dosages. The softening points of the nano-modified asphalt binders decrease approximately linearly as the bio-oil dosage increases.

**Fig 3:**
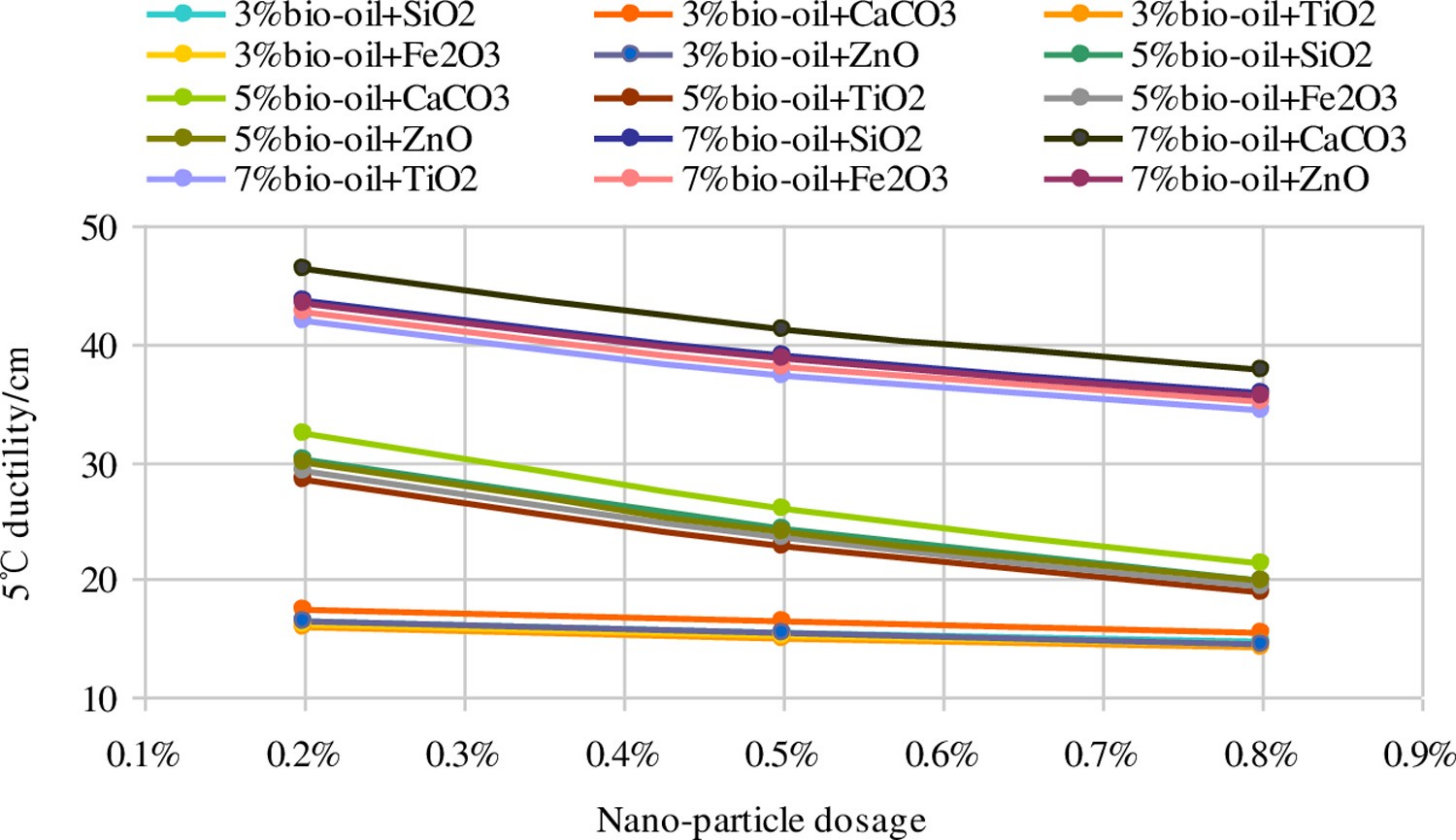
Trend of 5°C ductility with different nano-particle dosages.

Fig 3 displays the trend of 5°C ductility with different nano-particle dosages. The 5°C ductilities of the nano-modified bio-asphalt binders decrease linearly as the nano-particle dosages increase, indicating the adverse effect of the nano-particles on the low-temperature performance of the bio-asphalt binder. This occurs because the proportion of the dispersed phase of the bio-asphalt decreases with the addition of nano-particles, as previously mentioned.

This trend is not affected by the bio-oil dosage. However, although nano-particles have a disadvantageous effect on the low-temperature performance, the 5°C ductilities of the different nano-modified bio-asphalt binders are evidently better than that of the AH-70, showing the satisfactory low-temperature stability of the nano-modified bio-asphalt binders. Furthermore, the 5°C ductility of the nano-modified bio-asphalt binder using nano-CaCO_3_ is the highest, and those using SiO_2_ and TiO_2_ are the lowest. In addition, the 5°C ductilities of the nano-modified asphalt binders increase as the bio-oil dosage increase, and the trend is more evident with increased bio-oil dosages.

The 135°C dynamic viscosities of the nano-modified bio-asphalt binders increase slightly as the nano-particle dosages increase. However, the 135°C dynamic viscosities among all of the groups of experimental tests are always at a low level (lower than 1.0 Pa·s), meeting the Chinese specifications (≤ 3.0 Pa·s) [26].

### RTFOTs for evaluating the anti-aging performance of modified bio-asphalt binders

The mass loss, 25°C residual penetration ratio and 5°C ductility after aging, obtained via RTFOTs, are shown in Table 7.

**Table 7:** Test results of different asphalt binders in RTFOTs.

Type			Abbreviation	Mass loss (%)	Residual penetration ratio (%)	5°C ductility after aging (cm)
AH-70			BA	0.66	62.9	0
AH-70 +	3% bio-oil		BB-1	0.76	59.1	13.1
	5% bio-oil		BB-2	0.92	51.6	21.7
	7% bio-oil		BB-3	1.11	44.6	26.9
AH-70 +	3%bio-oil +	0.2%SiO_2_	BC-1	0.58	68.2	11.1
		0.5%SiO_2_	BC-2	0.54	70.2	10.6
		0.8%SiO_2_	BC-3	0.52	72.6	10.2
	5%bio-oil +	0.2%SiO_2_	BD-1	0.70	62.6	18.9
		0.5%SiO_2_	BD-2	0.66	64.8	15.5
		0.8%SiO_2_	BD-3	0.66	68.2	13.1
	7%bio-oil +	0.2%SiO_2_	BE-1	0.78	56.9	26.6
		0.5%SiO_2_	BE-2	0.76	59.6	24.6
		0.8%SiO_2_	BE-3	0.72	61.6	23.6
AH-70 +	3%bio-oil +	0.2%CaCO_3_	BF-1	0.70	60.1	11.1
		0.5%CaCO_3_	BF-2	0.66	61.2	10.6
		0.8%CaCO_3_	BF-3	0.62	62.1	10.1
	5%bio-oil +	0.2%CaCO_3_	BG-1	0.82	55.3	18.2
		0.5%CaCO_3_	BG-2	0.78	56.9	15.1
		0.8%CaCO_3_	BG-3	0.76	59.8	12.8
	7%bio-oil +	0.2%CaCO_3_	BH-1	0.92	48.8	26.6
		0.5%CaCO_3_	BH-2	0.88	51.5	24.2
		0.8%CaCO_3_	BH-3	0.80	53.3	22.6
AH-70 +	3%bio-oil +	0.2%TiO_2_	BI-1	0.60	66.2	9.8
		0.5%TiO_2_	BI-2	0.58	68.3	9.5
		0.8%TiO_2_	BI-3	0.56	69.6	9.1
	5%bio-oil +	0.2%TiO_2_	BJ-1	0.70	61.0	16.8
		0.5%TiO_2_	BJ-2	0.68	64.1	14.0
		0.8%TiO_2_	BJ-3	0.64	66.6	11.9
	7%bio-oil +	0.2%TiO_2_	BK-1	0.78	57.1	24.8
		0.5%TiO_2_	BK-2	0.74	61.2	22.8
		0.8%TiO_2_	BK-3	0.72	62.9	21.6
AH-70 +	3%bio-oil +	0.2%Fe_2_O_3_	BL-1	0.56	66.1	10.7
		0.5%Fe_2_O_3_	BL-2	0.54	68.1	10.2
		0.8%Fe_2_O_3_	BL-3	0.54	69.8	9.7
	5%bio-oil +	0.2%Fe_2_O_3_	BM-1	0.72	61.6	18.7
		0.5%Fe_2_O_3_	BM-2	0.70	64.1	15.3
		0.8%Fe_2_O_3_	BM-3	0.66	66.2	13.1
	7%bio-oil +	0.2%Fe_2_O_3_	BN-1	0.80	57.1	27.2
		0.5%Fe_2_O_3_	BN-2	0.76	60.2	24.6
		0.8%Fe_2_O_3_	BN-3	0.74	62.9	22.9
AH-70 +	3%bio-oil +	0.2%ZnO	BO-1	0.70	60.1	10.1
		0.5%ZnO	BO-2	0.66	61.2	9.6
		0.8%ZnO	BO-3	0.62	62.1	9.2
	5%bio-oil +	0.2%ZnO	BP-1	0.82	55.3	16.6
		0.5%ZnO	BP-2	0.78	57.9	13.6
		0.8%ZnO	BP-3	0.76	59.8	11.3
	7%bio-oil +	0.2%ZnO	BQ-1	0.92	48.8	24.8
		0.5%ZnO	BQ-2	0.88	51.5	22.6
		0.8%ZnO	BQ-3	0.80	53.3	21.1

**Fig 4:**
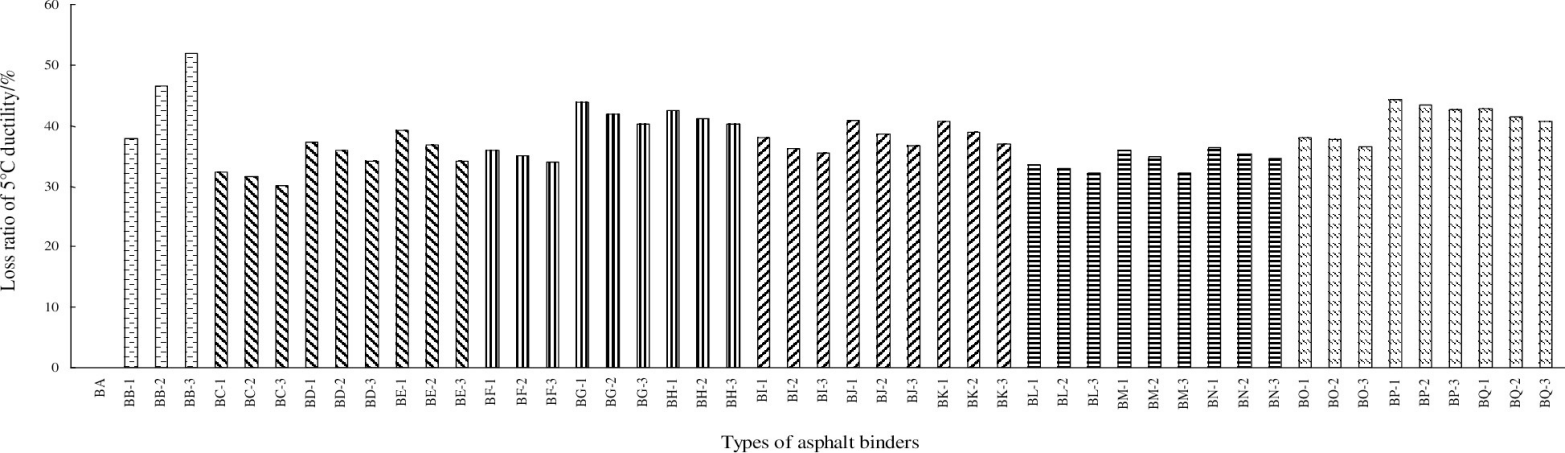
Loss ratios of the 5°C ductility.

As shown in Table 7, with increased nano-particle dosages, the mass losses of the nano-modified bio-asphalt binders decrease linearly and the residual penetration ratios increase accordingly, showing that nano-particles can improve the aging resistance of bio-asphalt binders. The enhancement effects from nano-SiO_2_ and nano-Fe_2_O_3_ are superior to those of the other nano-particles, and those of nano-CaCO_3_ and nano-ZnO are relatively weak. Moreover, after aging of the nano-modified bio-asphalt binders, the 5°C ductilities decrease as the nano-particle dosages increase, which may be owing to the negative effect of the nano-particles on these 5°C ductilities without aging. To explore further the effects of the nano-particles on the 5°C ductilities after aging, the loss ratios for the 5°C ductility ratio are calculated using Eq (1) and shown in Fig 4.

$$R_{a}=\left|D-D^{'}\right|/D\times 100\%$$ (1)

Where, *R*_*a*_ is the loss ratio of the 5°C ductility, *D* is the 5°C ductility without aging, and *D'* is the 5°C ductility after aging.

As shown in Fig 4, the loss ratios of the 5°C ductility of the nano-modified bio-asphalt binders decrease as the nano-particle dosages increase, indicating that the nano-particles provide the improved the aging resistance of bio-asphalt binders. This reduction trend of the loss ratio is more evident with increased bio-oil dosages. The above law is the most significant for nano-TiO_2_, and that for ZnO is the most insignificant. Moreover, on the whole, the loss ratios of the nano-modified bio-asphalt binders using nano-SiO_2_ are the lowest and those using nano-CaCO_3_ are the highest, showing the same law as the mass loss and the residual penetration ratio.

The mechanism of asphalt aging comprises the volatilization and transformation of the dispersed phase under the action of high temperatures and ultraviolet irradiation [34,35]. As previously mentioned, the proportion of the dispersed phase decreases with the addition of the nano-particles; thus the aging resistance of the nano-modified bio-asphalt binder is improved.

### Pavement properties of modified asphalt mixtures

**Fig 5:**
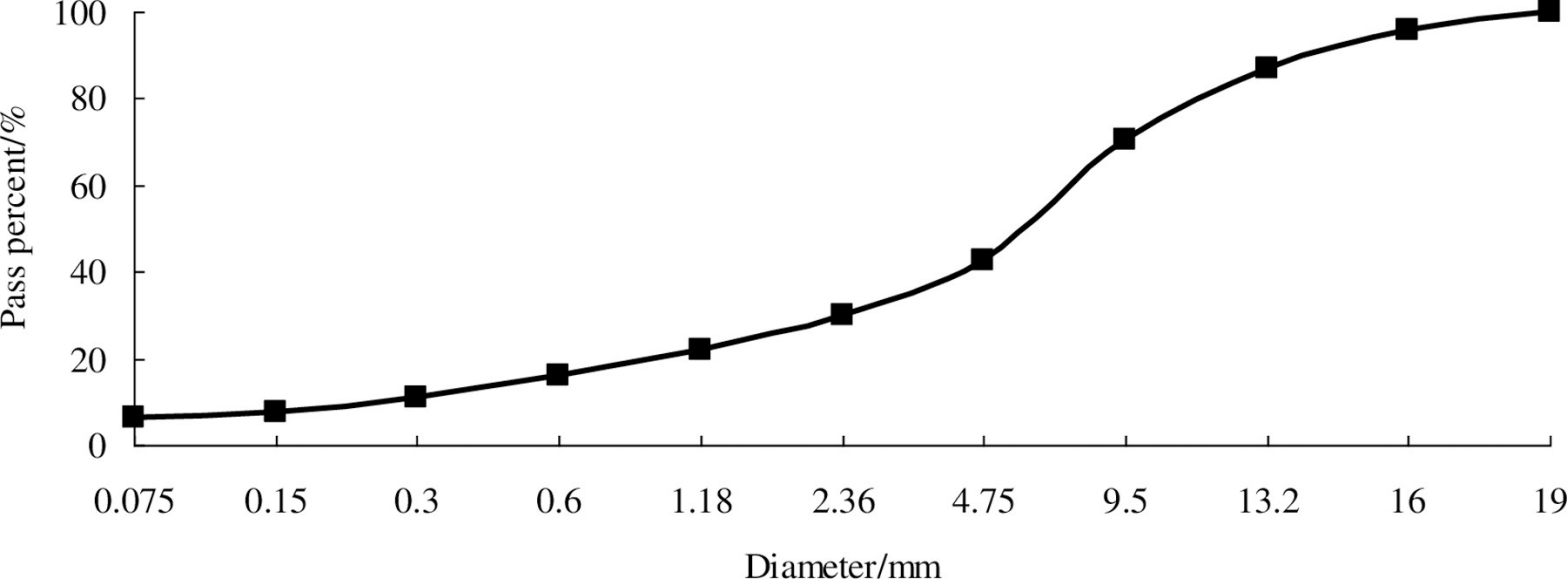
Aggregate gradation.

The aggregate gradation showed in Fig 5 is used in all types of asphalt mixtures. The dynamic stabilities, fracture strains and residual stabilities of the nano-modified bio-asphalt mixtures are listed in Table 8.

**Table 8:** Pavement properties of different asphalt mixtures.

Type			Dynamic stability (times·mm^-1^)	Fracture strain (*με*)	Residual stability (%)	Tensile strength ratio (%)
AH-70			2355	1682	86.2	86.3
AH-70 +	3% bio-oil		1021	3619	89.4	90.2
	5% bio-oil		862	5135	90.0	90.7
	7% bio-oil		786	6411	89.2	90.1
AH-70 +	3%bio-oil +	0.2%SiO_2_	2226	3198	89.8	90.5
		0.5%SiO_2_	2344	2945	89.2	90.3
		0.8%SiO_2_	2403	2893	88.3	89.4
	5%bio-oil +	0.2%SiO_2_	2077	4443	89.0	90.1
		0.5%SiO_2_	2177	4166	89.9	90.7
		0.8%SiO_2_	2216	4059	88.5	89.3
	7%bio-oil +	0.2%SiO_2_	1991	5853	89.9	90.7
		0.5%SiO_2_	2167	5519	89.5	90.4
		0.8%SiO_2_	2177	5480	89.4	90.3
AH-70 +	3%bio-oil +	0.2%CaCO_3_	2070	3217	88.1	89.0
		0.5%CaCO_3_	2180	2963	89.6	90.7
		0.8%CaCO_3_	2235	2934	88.6	89.8
	5%bio-oil +	0.2%CaCO_3_	1967	4617	88.4	89.5
		0.5%CaCO_3_	2024	4359	88.9	90.0
		0.8%CaCO_3_	2061	4299	88.4	89.6
	7%bio-oil +	0.2%CaCO_3_	1866	5900	88.1	89.2
		0.5%CaCO_3_	2016	5703	88.8	89.8
		0.8%CaCO_3_	2019	5639	88.4	89.5
AH-70 +	3%bio-oil +	0.2%TiO_2_	2092	3015	88.9	90.1
		0.5%TiO_2_	2203	2777	88.5	89.3
		0.8%TiO_2_	2259	2639	88.7	89.7
	5%bio-oil +	0.2%TiO_2_	1989	4243	89.9	90.8
		0.5%TiO_2_	2046	3985	88.6	89.6
		0.8%TiO_2_	2083	3866	89.9	91.1
	7%bio-oil +	0.2%TiO_2_	1909	5653	89.5	90.5
		0.5%TiO_2_	2037	5403	88.9	89.8
		0.8%TiO_2_	2036	5289	89.5	90.6
AH-70 +	3%bio-oil +	0.2%Fe_2_O_3_	2058	3102	89.1	90.0
		0.5%Fe_2_O_3_	2167	2857	89.0	90.1
		0.8%Fe_2_O_3_	2222	2798	88.9	89.8
	5%bio-oil +	0.2%Fe_2_O_3_	1946	4340	88.6	89.7
		0.5%Fe_2_O_3_	2013	4054	89.8	90.8
		0.8%Fe_2_O_3_	2049	3998	88.5	89.5
	7%bio-oil +	0.2%Fe_2_O_3_	1866	5735	89.0	89.9
		0.5%Fe_2_O_3_	2004	5429	89.7	90.6
		0.8%Fe_2_O_3_	2013	5366	89.0	90.1
AH-70 +	3%bio-oil +	0.2%ZnO	2035	3169	88.4	89.5
		0.5%ZnO	2142	2951	88.8	89.7
		0.8%ZnO	2196	2891	88.3	89.3
	5%bio-oil +	0.2%ZnO	1926	4425	88.3	89.5
		0.5%ZnO	1990	4177	89.7	90.6
		0.8%ZnO	2026	4100	89.0	90
	7%bio-oil +	0.2%ZnO	1844	5841	88.6	89.5
		0.5%ZnO	1981	5593	89.1	90.1
		0.8%ZnO	1990	5552	88.1	89.3

**Fig 6:**
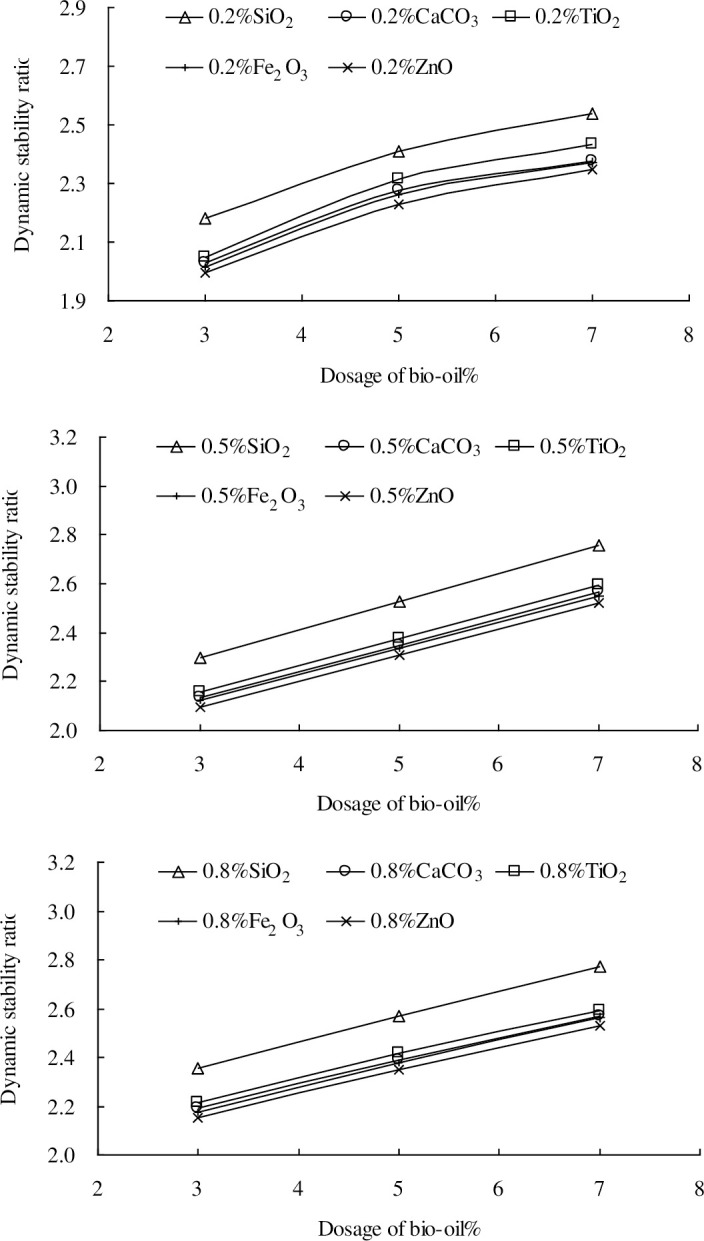
Dynamic stability ratio.

As shown in Table 8, it can be found that:

The effects of the five nano-particles on the dynamic stabilities of the nano-modified bio-asphalt mixtures are similar to those on the softening points of the nano-modified bio-asphalt binders. This indicates the positive effect of the five nano-particles on the high-temperature stabilities. To investigate the composite effect of the nano-particles and bio-oil on the high-temperature stability in a through manner, the dynamic stability ratios of the nano-modified bio-asphalt mixtures are calculated using Eq (2), as shown in Fig 6. The dynamic stability ratios reflect the strengthening effects of the different nano-particles on the dynamic stability.

$$DR_{i}=S_{i}/S_{\text{d}}$$ (2)

where, *DR*_*i*_ is the dynamic stability ratio of the nano-modified bio-asphalt mixtures with *i*% nano-particles (*i* = 0.2, 0.5 and 0.8), *S*_*i*_ is the dynamic stability of the nano-modified bio-asphalt mixtures with *i*% nano-materials, and *S*_*d*_ is the dynamic stability of the bio-asphalt mixtures without nano-particles.

As shown in Fig 6, the five nano-particles (at the different dosage levels) show a similar trend, i.e., that the strengthening effects on the dynamic stabilities increase linearly as the bio-oil dosage increased. Overall, nano-SiO_2_ is the best, and nano-ZnO is the worst (the differences of nano-TiO_2_, nano-CaCO_3_, nano-Fe_2_O_3_, nano-ZnO are limited).

**Fig 7:**
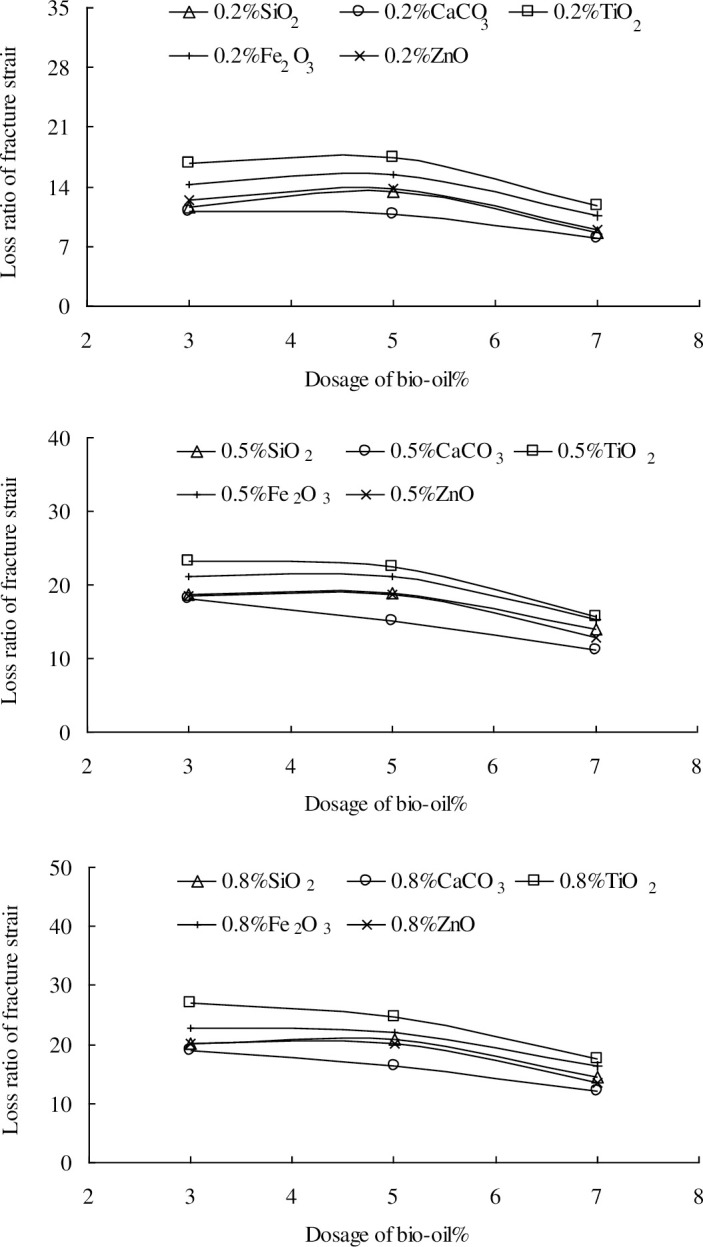
Loss ratio of fracture strain.

The fracture strains of the nano-modified bio-asphalt mixtures decrease as the nano-particle dosages increase, indicating the adverse effect of the nano-particles on the low-temperature crack resistance. The decreasing trends tend to flatten with increased nano-particle dosages. Moreover, the fracture strains of the nano-modified bio-asphalt mixtures are still evidently higher than those of the base asphalt mixture. To analyze the composite effect of the nano-particles and bio-oil on the low-temperature crack resistance of the nano-modified bio-asphalt mixtures further, the loss ratios of the fracture strains of the nano-modified bio-asphalt mixtures are calculated using Eq (3), as shown in Fig 7. The loss ratios reflect the effects of the different nano-particles.

$$R_{i}=\frac{\left|F_{i}^{'}-F\right|}{F}\times 100\%$$ (3)

where, *R*_*i*_ is the loss ratio of the fracture strain of the nano-modified bio-asphalt mixtures with *i*% nano-particles (*i* = 0.2, 0.5 and 0.8), *F* is the fracture strain of the bio-asphalt mixtures without nano-particles, and *F*_*i*_*'* is the fracture strain of the nano-modified bio-asphalt mixtures with *i*% nano-particles.

As shown in Fig 7, the negative effects of the nano-particles on the fracture strain decrease as bio-oil dosages increase. However, the trends of the loss ratios for the five nano-particles are identical. For nano-SiO_2_ and nano-CaCO_3_, this trend gradually tends to be linear with an increased bio-oil dosage. For nano-TiO_2_, nano-CaCO_3_, and nano-Fe_2_O_3_, the trend of the loss ratio is not evidently affected by the bio-oil dosage. It is relatively stable when the nano-particle dosage is lower than 0.5%. Overall, the negative effect of nano-SiO_2_ is the highest, and that of nano-CaCO_3_ is the lowest.

The residual stabilities of the different modified bio-asphalt mixtures are similar to each other but higher than that of the AH-70 base asphalt mixture, indicating the improved modified effectiveness of the nano-particles on the water stability.

The water stabilities of the different modified bio-asphalt mixtures are similar to each other but higher than that of the AH-70 base asphalt mixture, thereby demonstrating the positive modification effect of the asphalt binder on the water stability.

### Economic analysis

As previously mentioned, nano-particles can significantly improve the high-temperature performance of a bio-asphalt binder. However, relative to bio-oils and base asphalt binders, nano-particles are expensive. Hence, it is necessary to analyze the cost-effectiveness of a nano-modified bio-asphalt binder via comparison to AH-70. Table 9 shows the unit price of AH-70, bio-oil and nano-particles.

**Table 9:** Unit prices (¥/kg) of different materials.

AH-70	Bio-oil	SiO_2_	CaCO_3_	TiO_2_	Fe_2_O_3_	ZnO
4.0	1.1	40	20	40	40	30

As previously mentioned, the low-temperature performances and aging resistances of the nano-modified bio-asphalt binders are much higher than those of the AH-70, while the softening points have less difference. Moreover, according to the Chinese technical specification [26], the softening point of AH-70 must be higher than 43°C. Hence, we calculate the costs of nano-modified bio-asphalt binders whose softening points are higher than 43°C, as shown in Table 10. “¥” refers to Chinese Yuan (CNY).

**Table 10:** Cost of the nano-modified bio-asphalt binder.

Type			Cost (¥/kg)
AH-70			4.00
AH-70 +	3%bio-oil +	0.2%SiO_2_	3.99
		0.5%SiO_2_	4.11
		0.8%SiO_2_	4.23
	5%bio-oil +	0.2%SiO_2_	3.94
		0.5%SiO_2_	4.06
		0.8%SiO_2_	4.18
	7%bio-oil +	0.5%SiO_2_	4.00
		0.8%SiO_2_	4.12
AH-70 +	3%bio-oil +	0.5%CaCO_3_	4.01
		0.8%CaCO_3_	4.07
AH-70 +	3%bio-oil +	0.5%TiO_2_	4.11
		0.8%TiO_2_	4.23
	5%bio-oil +	0.8%TiO_2_	4.18
AH-70 +	3%bio-oil +	0.5%Fe_2_O_3_	4.11
		0.8%Fe_2_O_3_	4.23
AH-70 +	3%bio-oil +	0.5%ZnO	4.06
		0.8%ZnO	4.15

As shown in Table 10, compared to the AH-70, the cost of the nano-modified bio-asphalt binder is increased by 2.62% on average. Considering the improved low-temperature performance and aging resistance, it is reasonable to speculate that the nano-modified bio-asphalt binders present acceptable cost-effectiveness.

### Conclusions

To evaluate the modifying effects of nano-particles on bio-asphalt binders, the pavement performances and aging resistances of modified bio-asphalt binders with nano-SiO_2_, nano-TiO_2_, nano-CaCO_3_, nano-Fe_2_O_3_, and nano-ZnO are investigated in this study.

The high-temperature performances and aging resistances of the nano-modified bio-asphalt binders and mixtures are improved with increased nano-particle dosages, particularly for nano-SiO_2_. The modifying effect of nano-ZnO on the high-temperature performance and the modifying effect of nano-CaCO_3_ on the aging resistance are relatively weak.

The low-temperature performance of the nano-modified bio-asphalt is slightly weakened as compared to a bio-asphalt without nano-particles. The trends from nano-SiO_2_ and nano-CaCO_3_ are more sensitive than those of other nano-particles.

The effects of the nano-particles on the workable performance and water stability of modified bio-asphalt are insignificant.

## Supporting information

S1 File Data. This file includes all the test data of the asphalt binders and asphalt mixtures.
(DOC)
