# Supplementary material for: Investigating the pavement performance and aging resistance of modified bio-asphalt with nano-particles
Source: PLoS One. 2020 Sep 4;15(9):e0238817. doi: 10.1371/journal.pone.0238817 (PMC7473544; doi:10.1371/journal.pone.0238817)
Supplement: S1 File — This file includes all the test data of the asphalt binders and asphalt mixtures. (DOC) [file pone.0238817.s001.doc]

**Technical properties of different asphalt binders**

| Type | | | 25℃ penetration (0.1mm) | Softening point (℃) | 5℃ ductility (cm) | 135℃ viscosity (Pa.s) |
| --- | --- | --- | --- | --- | --- | --- |
| AH-70 | | | 67.4 | 47.4 | 0 | 0.680 |
| AH-70 + | 3% bio-oil | | 80.7 | 40.6 | 21.1 | 0.531 |
|  | 5% bio-oil | | 103.5 | 37.9 | 40.6 | 0.344 |
|  | 7% bio-oil | | -- | 35.8 | 55.9 | -- |
| AH-70 + | 3%bio-oil + | 0.2%SiO_2_ | 70.1 | 45.3 | 16.4 | 0.579 |
|  |  | 0.5%SiO_2_ | 67.1 | 47.7 | 15.5 | 0.638 |
|  |  | 0.8%SiO_2_ | 64.6 | 48.9 | 14.6 | 0.671 |
|  | 5%bio-oil + | 0.2%SiO_2_ | 82.8 | 43.7 | 30.1 | 0.568 |
|  |  | 0.5%SiO_2_ | 76.7 | 45.3 | 24.2 | 0.630 |
|  |  | 0.8%SiO_2_ | 70.1 | 46.1 | 19.9 | 0.645 |
|  | 7%bio-oil + | 0.2%SiO_2_ | 92.1 | 41.9 | 43.7 | 0.398 |
|  |  | 0.5%SiO_2_ | 87.6 | 44.1 | 38.9 | 0.462 |
|  |  | 0.8%SiO_2_ | 83.8 | 44.6 | 35.8 | 0.484 |
| AH-70 + | 3%bio-oil + | 0.2%CaCO_3_ | 78.2 | 42.1 | 17.3 | 0.546 |
|  |  | 0.5%CaCO_3_ | 73.1 | 44.4 | 16.3 | 0.602 |
|  |  | 0.8%CaCO_3_ | 68.7 | 45.5 | 15.3 | 0.633 |
|  | 5%bio-oil + | 0.2%CaCO_3_ | 92.4 | 40.6 | 32.4 | 0.536 |
|  |  | 0.5%CaCO_3_ | 85.6 | 42.2 | 26.0 | 0.594 |
|  |  | 0.8%CaCO_3_ | 78.2 | 42.9 | 21.4 | 0.608 |
|  | 7%bio-oil + | 0.2%CaCO_3_ | 102.8 | 39.3 | 46.3 | 0.375 |
|  |  | 0.5%CaCO_3_ | 97.8 | 41.0 | 41.1 | 0.436 |
|  |  | 0.8%CaCO_3_ | 93.5 | 41.5 | 37.8 | 0.456 |
| AH-70 + | 3%bio-oil + | 0.2%TiO_2_ | 75.7 | 42.6 | 15.8 | 0.565 |
|  |  | 0.5%TiO_2_ | 70.6 | 44.8 | 14.9 | 0.623 |
|  |  | 0.8%TiO_2_ | 66.5 | 46.0 | 14.1 | 0.655 |
|  | 5%bio-oil + | 0.2%TiO_2_ | 89.4 | 41.1 | 28.4 | 0.554 |
|  |  | 0.5%TiO_2_ | 82.8 | 42.6 | 22.8 | 0.615 |
|  |  | 0.8%TiO_2_ | 75.7 | 43.4 | 18.8 | 0.630 |
|  | 7%bio-oil + | 0.2%TiO_2_ | 99.5 | 39.6 | 41.8 | 0.388 |
|  |  | 0.5%TiO_2_ | 94.6 | 41.5 | 37.3 | 0.451 |
|  |  | 0.8%TiO_2_ | 90.5 | 41.9 | 34.3 | 0.472 |
| AH-70 + | 3%bio-oil + | 0.2%Fe_2_O_3_ | 71.2 | 41.9 | 16.1 | 0.570 |
|  |  | 0.5%Fe_2_O_3_ | 66.1 | 44.1 | 15.2 | 0.628 |
|  |  | 0.8%Fe_2_O_3_ | 62.5 | 45.2 | 14.3 | 0.660 |
|  | 5%bio-oil + | 0.2%Fe_2_O_3_ | 84.0 | 40.4 | 29.2 | 0.559 |
|  |  | 0.5%Fe_2_O_3_ | 77.9 | 42.0 | 23.5 | 0.620 |
|  |  | 0.8%Fe_2_O_3_ | 71.2 | 42.6 | 19.3 | 0.635 |
|  | 7%bio-oil + | 0.2%Fe_2_O_3_ | 93.5 | 38.9 | 42.7 | 0.392 |
|  |  | 0.5%Fe_2_O_3_ | 88.9 | 40.8 | 38.0 | 0.455 |
|  |  | 0.8%Fe_2_O_3_ | 85.1 | 41.3 | 35.0 | 0.476 |
| AH-70 + | 3%bio-oil + | 0.2%ZnO | 72.6 | 41.4 | 16.3 | 0.544 |
|  |  | 0.5%ZnO | 67.6 | 43.6 | 15.4 | 0.600 |
|  |  | 0.8%ZnO | 63.8 | 44.7 | 14.5 | 0.631 |
|  | 5%bio-oil + | 0.2%ZnO | 85.8 | 39.9 | 29.8 | 0.534 |
|  |  | 0.5%ZnO | 79.5 | 41.7 | 24.0 | 0.592 |
|  |  | 0.8%ZnO | 72.6 | 42.2 | 19.7 | 0.606 |
|  | 7%bio-oil + | 0.2%ZnO | 95.4 | 38.1 | 43.4 | 0.374 |
|  |  | 0.5%ZnO | 90.8 | 40.3 | 38.6 | 0.434 |
|  |  | 0.8%ZnO | 86.8 | 41.0 | 35.6 | 0.455 |

**Test results of different asphalt binders in RTFOTs**

| Type | | | Abbreviation | Mass loss (%) | Residual penetration ratio (%) | 5℃ ductility after aging (cm) |
| --- | --- | --- | --- | --- | --- | --- |
| AH-70 | | | BA | 0.66 | 62.9 | 0 |
| AH-70 + | 3% bio-oil | | BB-1 | 0.76 | 59.1 | 13.1 |
|  | 5% bio-oil | | BB-2 | 0.92 | 51.6 | 21.7 |
|  | 7% bio-oil | | BB-3 | 1.11 | 44.6 | 26.9 |
| AH-70 + | 3%bio-oil + | 0.2%SiO_2_ | BC-1 | 0.58 | 68.2 | 11.1 |
|  |  | 0.5%SiO_2_ | BC-2 | 0.54 | 70.2 | 10.6 |
|  |  | 0.8%SiO_2_ | BC-3 | 0.52 | 72.6 | 10.2 |
|  | 5%bio-oil + | 0.2%SiO_2_ | BD-1 | 0.70 | 62.6 | 18.9 |
|  |  | 0.5%SiO_2_ | BD-2 | 0.66 | 64.8 | 15.5 |
|  |  | 0.8%SiO_2_ | BD-3 | 0.66 | 68.2 | 13.1 |
|  | 7%bio-oil + | 0.2%SiO_2_ | BE-1 | 0.78 | 56.9 | 26.6 |
|  |  | 0.5%SiO_2_ | BE-2 | 0.76 | 59.6 | 24.6 |
|  |  | 0.8%SiO_2_ | BE-3 | 0.72 | 61.6 | 23.6 |
| AH-70 + | 3%bio-oil + | 0.2%CaCO_3_ | BF-1 | 0.70 | 60.1 | 11.1 |
|  |  | 0.5%CaCO_3_ | BF-2 | 0.66 | 61.2 | 10.6 |
|  |  | 0.8%CaCO_3_ | BF-3 | 0.62 | 62.1 | 10.1 |
|  | 5%bio-oil + | 0.2%CaCO_3_ | BG-1 | 0.82 | 55.3 | 18.2 |
|  |  | 0.5%CaCO_3_ | BG-2 | 0.78 | 56.9 | 15.1 |
|  |  | 0.8%CaCO_3_ | BG-3 | 0.76 | 59.8 | 12.8 |
|  | 7%bio-oil + | 0.2%CaCO_3_ | BH-1 | 0.92 | 48.8 | 26.6 |
|  |  | 0.5%CaCO_3_ | BH-2 | 0.88 | 51.5 | 24.2 |
|  |  | 0.8%CaCO_3_ | BH-3 | 0.80 | 53.3 | 22.6 |
| AH-70 + | 3%bio-oil + | 0.2%TiO_2_ | BI-1 | 0.60 | 66.2 | 9.8 |
|  |  | 0.5%TiO_2_ | BI-2 | 0.58 | 68.3 | 9.5 |
|  |  | 0.8%TiO_2_ | BI-3 | 0.56 | 69.6 | 9.1 |
|  | 5%bio-oil + | 0.2%TiO_2_ | BJ-1 | 0.70 | 61.0 | 16.8 |
|  |  | 0.5%TiO_2_ | BJ-2 | 0.68 | 64.1 | 14.0 |
|  |  | 0.8%TiO_2_ | BJ-3 | 0.64 | 66.6 | 11.9 |
|  | 7%bio-oil + | 0.2%TiO_2_ | BK-1 | 0.78 | 57.1 | 24.8 |
|  |  | 0.5%TiO_2_ | BK-2 | 0.74 | 61.2 | 22.8 |
|  |  | 0.8%TiO_2_ | BK-3 | 0.72 | 62.9 | 21.6 |
| AH-70 + | 3%bio-oil + | 0.2%Fe_2_O_3_ | BL-1 | 0.56 | 66.1 | 10.7 |
|  |  | 0.5%Fe_2_O_3_ | BL-2 | 0.54 | 68.1 | 10.2 |
|  |  | 0.8%Fe_2_O_3_ | BL-3 | 0.54 | 69.8 | 9.7 |
|  | 5%bio-oil + | 0.2%Fe_2_O_3_ | BM-1 | 0.72 | 61.6 | 18.7 |
|  |  | 0.5%Fe_2_O_3_ | BM-2 | 0.70 | 64.1 | 15.3 |
|  |  | 0.8%Fe_2_O_3_ | BM-3 | 0.66 | 66.2 | 13.1 |
|  | 7%bio-oil + | 0.2%Fe_2_O_3_ | BN-1 | 0.80 | 57.1 | 27.2 |
|  |  | 0.5%Fe_2_O_3_ | BN-2 | 0.76 | 60.2 | 24.6 |
|  |  | 0.8%Fe_2_O_3_ | BN-3 | 0.74 | 62.9 | 22.9 |
| AH-70 + | 3%bio-oil + | 0.2%ZnO | BO-1 | 0.70 | 60.1 | 10.1 |
|  |  | 0.5%ZnO | BO-2 | 0.66 | 61.2 | 9.6 |
|  |  | 0.8%ZnO | BO-3 | 0.62 | 62.1 | 9.2 |
|  | 5%bio-oil + | 0.2%ZnO | BP-1 | 0.82 | 55.3 | 16.6 |
|  |  | 0.5%ZnO | BP-2 | 0.78 | 57.9 | 13.6 |
|  |  | 0.8%ZnO | BP-3 | 0.76 | 59.8 | 11.3 |
|  | 7%bio-oil + | 0.2%ZnO | BQ-1 | 0.92 | 48.8 | 24.8 |
|  |  | 0.5%ZnO | BQ-2 | 0.88 | 51.5 | 22.6 |
|  |  | 0.8%ZnO | BQ-3 | 0.80 | 53.3 | 21.1 |

**Pavement properties of different asphalt mixtures**

| Type | | | Dynamic stability (times·mm^-1^) | Fracture strain (*με*) | Residual stability (%) | Tensile strength ratio (%) |
| --- | --- | --- | --- | --- | --- | --- |
| AH-70 | | | 2355 | 1682 | 86.2 | 86.3 |
| AH-70 + | 3% bio-oil | | 1021 | 3619 | 89.4 | 90.2 |
|  | 5% bio-oil | | 862 | 5135 | 90.0 | 90.7 |
|  | 7% bio-oil | | 786 | 6411 | 89.2 | 90.1 |
| AH-70 + | 3%bio-oil + | 0.2%SiO_2_ | 2226 | 3198 | 89.8 | 90.5 |
|  |  | 0.5%SiO_2_ | 2344 | 2945 | 89.2 | 90.3 |
|  |  | 0.8%SiO_2_ | 2403 | 2893 | 88.3 | 89.4 |
|  | 5%bio-oil + | 0.2%SiO_2_ | 2077 | 4443 | 89.0 | 90.1 |
|  |  | 0.5%SiO_2_ | 2177 | 4166 | 89.9 | 90.7 |
|  |  | 0.8%SiO_2_ | 2216 | 4059 | 88.5 | 89.3 |
|  | 7%bio-oil + | 0.2%SiO_2_ | 1991 | 5853 | 89.9 | 90.7 |
|  |  | 0.5%SiO_2_ | 2167 | 5519 | 89.5 | 90.4 |
|  |  | 0.8%SiO_2_ | 2177 | 5480 | 89.4 | 90.3 |
| AH-70 + | 3%bio-oil + | 0.2%CaCO_3_ | 2070 | 3217 | 88.1 | 89.0 |
|  |  | 0.5%CaCO_3_ | 2180 | 2963 | 89.6 | 90.7 |
|  |  | 0.8%CaCO_3_ | 2235 | 2934 | 88.6 | 89.8 |
|  | 5%bio-oil + | 0.2%CaCO_3_ | 1967 | 4617 | 88.4 | 89.5 |
|  |  | 0.5%CaCO_3_ | 2024 | 4359 | 88.9 | 90.0 |
|  |  | 0.8%CaCO_3_ | 2061 | 4299 | 88.4 | 89.6 |
|  | 7%bio-oil + | 0.2%CaCO_3_ | 1866 | 5900 | 88.1 | 89.2 |
|  |  | 0.5%CaCO_3_ | 2016 | 5703 | 88.8 | 89.8 |
|  |  | 0.8%CaCO_3_ | 2019 | 5639 | 88.4 | 89.5 |
| AH-70 + | 3%bio-oil + | 0.2%TiO_2_ | 2092 | 3015 | 88.9 | 90.1 |
|  |  | 0.5%TiO_2_ | 2203 | 2777 | 88.5 | 89.3 |
|  |  | 0.8%TiO_2_ | 2259 | 2639 | 88.7 | 89.7 |
|  | 5%bio-oil + | 0.2%TiO_2_ | 1989 | 4243 | 89.9 | 90.8 |
|  |  | 0.5%TiO_2_ | 2046 | 3985 | 88.6 | 89.6 |
|  |  | 0.8%TiO_2_ | 2083 | 3866 | 89.9 | 91.1 |
|  | 7%bio-oil + | 0.2%TiO_2_ | 1909 | 5653 | 89.5 | 90.5 |
|  |  | 0.5%TiO_2_ | 2037 | 5403 | 88.9 | 89.8 |
|  |  | 0.8%TiO_2_ | 2036 | 5289 | 89.5 | 90.6 |
| AH-70 + | 3%bio-oil + | 0.2%Fe_2_O_3_ | 2058 | 3102 | 89.1 | 90.0 |
|  |  | 0.5%Fe_2_O_3_ | 2167 | 2857 | 89.0 | 90.1 |
|  |  | 0.8%Fe_2_O_3_ | 2222 | 2798 | 88.9 | 89.8 |
|  | 5%bio-oil + | 0.2%Fe_2_O_3_ | 1946 | 4340 | 88.6 | 89.7 |
|  |  | 0.5%Fe_2_O_3_ | 2013 | 4054 | 89.8 | 90.8 |
|  |  | 0.8%Fe_2_O_3_ | 2049 | 3998 | 88.5 | 89.5 |
|  | 7%bio-oil + | 0.2%Fe_2_O_3_ | 1866 | 5735 | 89.0 | 89.9 |
|  |  | 0.5%Fe_2_O_3_ | 2004 | 5429 | 89.7 | 90.6 |
|  |  | 0.8%Fe_2_O_3_ | 2013 | 5366 | 89.0 | 90.1 |
| AH-70 + | 3%bio-oil + | 0.2%ZnO | 2035 | 3169 | 88.4 | 89.5 |
|  |  | 0.5%ZnO | 2142 | 2951 | 88.8 | 89.7 |
|  |  | 0.8%ZnO | 2196 | 2891 | 88.3 | 89.3 |
|  | 5%bio-oil + | 0.2%ZnO | 1926 | 4425 | 88.3 | 89.5 |
|  |  | 0.5%ZnO | 1990 | 4177 | 89.7 | 90.6 |
|  |  | 0.8%ZnO | 2026 | 4100 | 89.0 | 90 |
|  | 7%bio-oil + | 0.2%ZnO | 1844 | 5841 | 88.6 | 89.5 |
|  |  | 0.5%ZnO | 1981 | 5593 | 89.1 | 90.1 |
|  |  | 0.8%ZnO | 1990 | 5552 | 88.1 | 89.3 |
